# Novel design for a phase IIa placebo-controlled, double-blind randomized withdrawal study to evaluate the safety and efficacy of CNV1014802 in patients with trigeminal neuralgia

**DOI:** 10.1186/1745-6215-14-402

**Published:** 2013-11-23

**Authors:** Joanna M Zakrzewska, Joanne Palmer, Dominik A Ettlin, Mark Obermann, Gerard MP Giblin, Valerie Morisset, Simon Tate, Kevin Gunn

**Affiliations:** 1 Facial pain unit, Division of Diagnostic, Surgical and Medical Sciences, Eastman Dental Hospital, UCLH NHS Foundation Trust/University College London, 256 Gray’s Inn Road, WC1X 8LD, London, UK; 2 Convergence Pharmaceuticals Ltd, Maia Building, Babraham Research Campus, CB22 3AT, Cambridge, UK; 3 Interdiscipilinary Orofacial Pain Unit, Clinic of Masticatory Disorders, Removable Prosthodontics, Geriatric and Special Care Dentistry, Center of Dental Medicine, University of Zurich, Plattenstrasse 11, CH-8032, Zurich, Switzerland; 4 Department of Neurology and German Headache Center, University of Duisburg-Essen, Hufelandstr. 55, 45147, Essen, Germany

**Keywords:** Trigeminal neuralgia, Double-blind randomized withdrawal study, Sodium channel blocker, Neuropathic pain

## Abstract

**Background:**

Trigeminal neuralgia (TN) is a rare severe unilateral facial pain condition. Current guidelines in trigeminal neuralgia management recommend sodium channel blockers – carbamazepine or oxcarbazepine – as the first-line treatment. However, the currently available drugs are often associated with poor tolerability resulting in sub-optimal pain control. CNV1014802 is a novel sodium channel blocker that is being assessed in the treatment of trigeminal neuralgia. Due to the severity of the condition, it is not ethical to conduct a traditional placebo-controlled randomized controlled trial. It is also difficult to use an active control such as carbamazepine, the current gold standard, because of its complex pharmacology and potential for drug interactions.

**Methods/Design:**

The trial uses a randomized withdrawal design to assess efficacy in this rare condition. There is a 21-day open-label phase followed by a randomized 28-day placebo-controlled phase for responders. Thirty patients will be randomized. The primary outcome measure will be pain relief, but secondary measures of quality of life will be of significant importance given the effect of this condition on activities of daily living. Safety and adverse event endpoints are described.

**Discussion:**

There have been very few well-controlled, randomized, placebo-controlled studies in trigeminal neuralgia, and the majority of drugs have had other primary uses. Due to the severity of the pain, minimizing the time a patient is administered placebo was a key factor in designing this study. This study will not only provide data on the efficacy of CNV1014802 in trigeminal neuralgia, but will also provide information on the effectiveness and acceptability of a novel trial design in trigeminal neuralgia.

**Trial registration:**

Trial number NCT01540630

## Background

Trigeminal neuralgia (TN) is an uncommon episodic severe facial pain condition with an incidence of 4.0 to 4.7 per 100,000 persons per year [1,2]. Trigeminal neuralgia can appear at any age, but disease onset is over 40 years of age in over 90% of cases with peak onset between 50 and 70 years of age [2,3]. However, recent data from primary care practice data validated by experts suggest an incidence rate of 12.6 per 100,000 person years with a mean age at diagnosis of 51.5 (SD 17.6) with a female predominance 71% [4]. The classification system of the International Headache Association aims at establishing TN diagnostic criteria based on etiology [5]. Yet, there is a problem in distinguishing primary and secondary TN, because unless a patient with normal neuroimaging comes to an operation, it remains unclear if his/her TN is caused by a vascular compression. To adjust for this diagnostic uncertainty, the term “classical” neuralgia is currently used for cases with normal neuroimaging and potential compression of the proximal trigeminal nerve root by a vascular loop. Classical TN is the most common TN type, and it is thought that secondary demyelination, probably mediated by microvascular ischemic damage, results in a lowered excitability threshold of affected neurons. This promotes inappropriate ectopic generation of spontaneous nerve impulses together with abnormal nonsynaptic ephatic transmission to adjacent neurons [6,7].

Unlike many other neuropathic pains, TN results in recurrent paroxysms of short-lasting but very severe pain in the distribution of one or more branches of the trigeminal nerve. In patients with the classical type, the attacks come on suddenly, last up to 2 min and disappear suddenly. Between attacks, patients are usually asymptomatic, although there may be a slight after pain [8]. Numerous attacks occur a day, but currently there are no studies detailing how many paroxysms occur a day. In a recent RCT of botulinum toxin A in 42 TN patients, the frequency of paroxysms was measured, and the average was 20 a day, but the range was 4–100 [9]. Patients also report that these can be so frequent that it seems like one long attack. The attacks can vary in severity and be described as shooting, electric shocks scoring maximum scores on scales or just “twinges.” These attacks can then go on for weeks or months. Especially in the early stages of the disorder, it is very common for the attacks to stop completely and for patients to have weeks, months or even years of no pain [10]. However, over time the remission periods get shorter. Currently, there are no natural history studies or known prognostic factors to help determine how long these periods can be, although Taylor et al. [11] showed that carbamazepine became less effective with time. This may be due to the natural course of the disease, but may also be vested in the autoinduction pharmacological properties of carbamazepine itself. Pain is provoked by light touch activities, e.g., washing, eating, talking and cold winds, but spontaneous attacks of pain also occur. One study that measured the occurrence of evoked or spontaneous pain suggested that oxcarbazepine was more effective at reducing the former [12]. Although attacks of pain occur at night, these are less common. Thus, even when pain free, patients live in fear of pain return. These features therefore make it difficult to evaluate the effect of treatments. The diagnostic criteria for TN most frequently used are those of the International Headache Society, the International Classification of Headache Disorders (ICHD) [13], and these have now been updated with clearer specification of the variants of TN, i.e., those patients who may have in addition to the shooting stabbing pain a more prolonged pain, and these are thought to have a potentially different pathophysiology [14].

Current guidelines in trigeminal neuralgia management recommend sodium channel blockers such as carbamazepine or oxcarbazepine as the first-line treatment for pain control. However, these agents are often poorly tolerated [15] and often require lengthy dose escalation, resulting in sub-optimal efficacy, and some result in significant drug interactions and require careful monitoring. Other potential second-line therapies include lamotrigine, pregabalin, gabapentin and baclofen; there is limited evidence to support the use of these agents [15,16]. Unlike other medications used in chronic pain, these drugs often result in complete pain relief, not just 50%, especially in the earlier stages of the disorder.

All the drugs used to date have been initially developed for other uses, principally epilepsy, so dosage scales have had to be adapted. A review of RCTs and Cochrane systematic reviews (SR) shows that the major side effects reported from antiepileptic drugs (AED) were drowsiness from 100–4%, dizziness or vertigo 47–3%, gastrointestinal (GI) 57 to- 8%, mood changes 2%, dry mouth or taste change 4–2%, and headaches 4%. The SR on carbamazepine (CBZ) [17] reported 40–60% would have side effects. However, no study provides details on how these events were measured or quantified. Studies in healthy volunteers do show that AED drugs, especially the older ones such as CBZ, do result in cognitive impairment, although this is generally modest but can have clinical significance [18]. Memory, especially those tasks with an attentional component, is reduced when on CBZ [19,20]. However, it also needs to be noted that pain has an impact on cognitive function [21].

Thus, a well-tolerated sodium channel blocker that can be administered at an effective dose with no titration and good tolerability may address some of the unmet needs of this patient group.

CNV1014802 is a peripherally and centrally acting agent that inhibits sodium channels in a state-dependent fashion. CNV1014802 shows selectivity for the Nav1.7 subtype over the other subtypes tested (Nav1.1, Nav1.2, Nav1.3, Nav1.5, Nav1.6 and TTX-R), for both the resting and depolarized states. The greater block of Nav1.7 is particularly enhanced at concentrations below 1 μM, where the free exposure in animal models and human clinical doses lies.

In addition, the amount of block by CNV1014802 increases significantly and in a similar way with the frequency of stimulation for Nav1.7, Nav1.2 and Nav1.6. Combining all aspects of the pharmacology of CNV101802 at sodium channels, the block is more activity-driven at Nav1.2 and Nav1.6 than it is at Nav1.7, where it is substantial even at lower levels of activity of the channel. The block at Nav1.5 and TTX-R is significantly weaker.

Consistent with its mechanism of action, CNV1014802 will preferentially target and inhibit higher frequencies of firing (from 10 Hz onwards) that are attained following noxious stimuli or occur in chronic pain conditions or during seizure activity.

CNV1014802 has also been shown to selectively and reversibly inhibit the monoamine oxidase (MAO)-B enzyme, with no effect on MAO-A. One hundred percent inhibition of MAO-B was achieved with doses of 75 mg and above in the clinic studies.

A genetic substrate for neuropathic pain is an accepted hypothesis in the scientific community. Recently, sodium channel gene mutations causing cell hyperexcitability have been identified in groups of patients with painful neuropathy [22,23]. Calcium channelopathies have also been linked to migraine and epilepsy [24]. Given the importance of sodium and calcium channels in the generation, propagation and plasticity of pain signals, it is proposed to genotype five sodium channel (Nav1.1, Nav1.2, Nav1.3, Nav1.6 and Nav1.7) and two calcium channel (Cav2.2 and Cav2.1) genes in all patients entering the study to explore whether mutations in these genes are present in TN and whether these are related to response to treatment with CNV1014802.

CNV1014802 has completed extensive phase I studies with single and repeated doses in 166 healthy volunteers.

Moore et al. [25] in their analysis of analgesic drugs propose that high failure rates of drugs must be expected especially in chronic pain, that a radical re-think is necessary in the design of analgesic trials and that an enhancement enriched randomized withdrawal design may be the way forward, but currently they are poorly understood in part because few have been carried out.

Thus, the aim of this study is to design and test a protocol to evaluate the efficacy of a new sodium channel blocker, CNV1014802, in TN, which takes into account the difficulties encountered in previous trials in this area. An additional aim, if study participants agree, is genotyping for possible sodium and calcium channelopathies. The protocol was designed and approved prior to the SPIRIT 2013 statement, but will try to adhere to the published check list [26].

## Methods and design

A review of the literature was first carried out and showed that clinical trials in TN are challenging, and many of the published trials in this disorder have recruited low numbers of patients and have used variable designs and outcome measures, resulting in inconclusive outcomes [17,27-29]. Traditional placebo-controlled studies (crossover or parallel group) are difficult to run in this condition, as significant numbers of patients will be exposed to placebo for an extended duration. Using active controls is difficult as the gold standard drug carbamazepine is metabolized through the liver and so takes time to be eliminated and for the liver enzymes to return to normal function, thus necessitating extended washout periods. Because of the severe nature of the pain, patients are unlikely to accept extended periods of no treatment or use of placebos. Two-stage enhancement enriched randomized withdrawal designs (EERW) have recently been proposed as an alternative strategy for determining the effectiveness of analgesic drugs [30,31]. Although bias will occur as only responders are enrolled into the randomized part of the trial and they may also guess whether they are on the active drug or not, it does offer the patients a rapid termination and return to previous medication. Response to the open label part of the study also allows any adjustments to be made to the dosage and to evaluate whether the drug has any potential in the individual patient. A recent study using levetiracetam for TN showed poor efficacy in the open label phase, so the randomized trial was not started [32]. With a disorder that results in unexpected remission periods, it is essential to ensure that at the end of the trial it is established that the disease has not gone into remission.

Outcome measures were chosen according to the recently published recommendations from the International Association for the Study of Pain committee [33], which suggest the following areas should be assessed using psychometrically tested tools: pain intensity, physical and emotional functioning, participants global improvement and satisfaction, symptoms and adverse events, and participants disposition. Trigeminal neuralgia is unusual in that it is considered a chronic pain, but the pain is very clearly episodic. Attacks can vary in number and anecdotally patients have reported that a significant outcome is to have a reduction in pain attacks even if their intensity remains high. In an RCT of use of lamotrigine in TN, a composite score was obtained that included the number of bursts of pain, i.e., not individual stabs of pain but paroxysms of pain (none, 1–3, 4–7, 8–12, 13–20, over 20), severity of pain (no pain, mild, moderate, severe) and degree of pain relief (complete, good, moderate, slight, none) [34]. Thus, the number of paroxysms daily would also be used as an outcome measure, although there are no data on the number of attacks expected, but a 50% reduction would be considered successful. The pain intensity of each paroxysm is assessed on an 11-point numerical rating scale (PI-NRS). The Brief Pain Inventory-Facial (BPI-Facial), which is a validated 18-item rating scale for facial pain in a TN population [35], is also included as a composite assessment of efficacy, which includes the impact of pain on quality of life.

Special emphasis will be placed on adverse events as drugs such as carbamazepine are highly effective, but their tolerability is very poor, thus limiting their use. A scale that has been developed and tested in large numbers of patients with epilepsy is the adverse events profile (AEP) by Baker et al. [36,37]. This would provide more quantifiable data than have previously been reported.

Although measuring cognitive function is complex and it has been suggested that self-administered scales may have a much stronger relationship to participants’ mood rather than their objectively measured cognitive performance, some attempt will be made to measure this using the Medical Outcomes Study-Cognitive Scale (MOS-Cog) [38,39].

Genotyping is not mandatory for patients participating in the study.

The trial was designed by the chief investigator (JZ) and the Convergence team with protocol review by two outside neurologists with experience in this disorder and later by two of the authors (MO; DE) Additional file 1. A brief summary of the trial was presented at a UK Trigeminal Neuralgia support group meeting to determine whether patients would take part in the study and whether the correct outcome measures were chosen. Expert patients were asked to comment on the patient information sheet, consent form and diary card. The study has been approved by regulatory authorities and ethics committees in 11 countries: the UK, Germany, Switzerland, Estonia, France, Italy, Latvia, Lithuania, Romania, South Africa and Spain. The names of the committees are to be found in the Additional file 2, Ethics Committees. Although it is anticipated that patients will be referred in from the community, the study will take place in secondary care centers, which have a special interest in headache and facial pain.

The trial was designed to use an initial dose of 150 mg three times daily with the flexibility to use a higher dose regimen of 350 mg twice daily if appropriate. A review of efficacy data after the first ten patients had completed the open label study was planned to review efficacy and tolerability. If these were acceptable, as judged by a data monitoring committee, then the trial would continue to completion without changing the dose. All investigators and their teams met for 1 day to undertake training and review the protocol, and the operational practicalities of the study including site selection, monitoring and data management were taken over by a contract research organization (CRO).

The outline protocol is available at http://www.clintrials.gov, trial no. NCT01540630, and gained ethical approval in the UK in September 2011, then subsequently in all the other participating countries. A final harmonized version of the protocol, incorporating all country-specific minor amendments, was available in August 2013.

**Figure 1:**
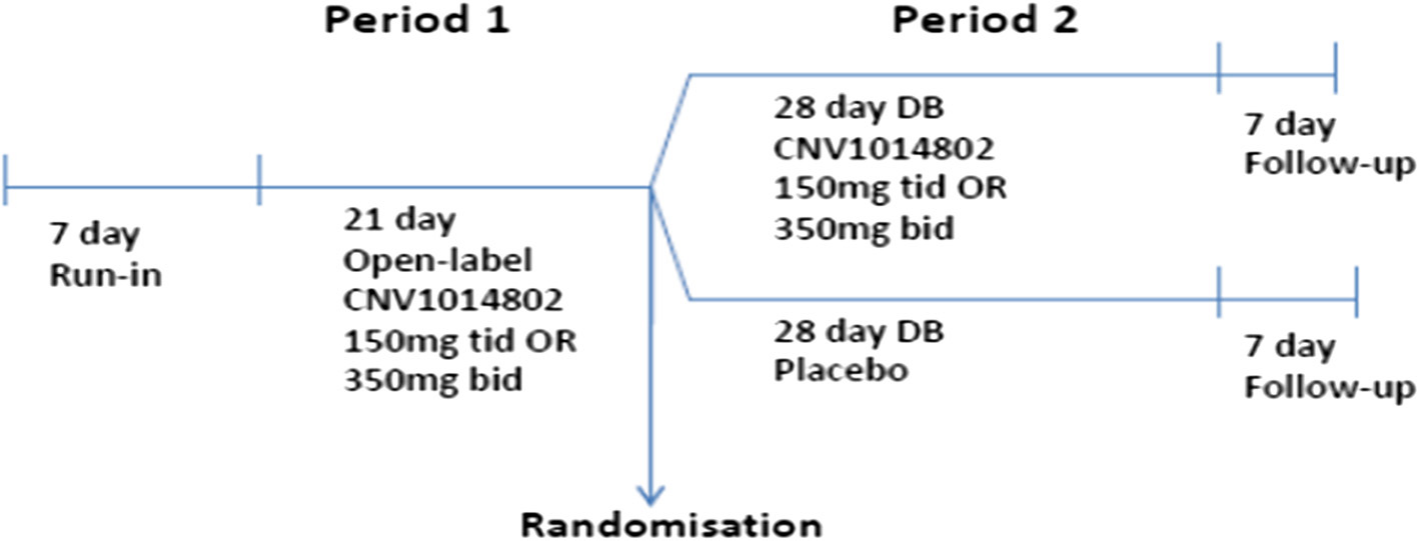
Design of the study.

A double-blind, randomized withdrawal study comparing CNV1014802 with placebo in patients with TN who have successfully responded to CNV1014802 in an initial open-label phase was designed to take into account all the difficulties highlighted in the Methods section. Figure 1 shows the overall design of the study.

### Design

Patients participate in an initial open-label treatment period of 21 days of CNV1014802 using 150 mg three times daily. Responders are then randomized to 28 days of CNV1014802 150 mg three times daily or placebo.

A responder at the end of the open-label period was defined as a patient with one of the following:

• A 30% or more decrease in the total number of paroxysms over the last 7 days of the open-label phase as compared to the total number recorded in the 7-day baseline phase (day -7 to -1 prior to start of study medication)

• A 30% reduction in the mean severity of pain experienced during the paroxysm over the last 7 days of the open-label phase as compared to the total number recorded in the 7-day baseline phase (day -7 to -1 prior to start of study medication)

• A Patient Global Improvement of Change rating of much improved/very much improved

### Randomization and allocation concealment mechanism

In the initial open-label treatment period, all patients will receive CNV1014802.

Patients will be randomized to CNV1014802 or placebo, in a 1:1 ratio, in accordance with the randomization schedule. The randomization schedule will be generated by the CRO using the PROC PLAN procedure of SAS®, a validated computer system. Eligible patients will be randomized into the study on day 21. The randomization schedule will be stratified by whether the patient is on existing pain medication (adjunct) or not (monotherapy). Equal numbers of patient on adjunct and monotherapy are not required.

### Outcome measures

The primary outcome measure of efficacy is the number of treatment failures on CNV1014802 vs. number of treatment failures on placebo throughout the double-blind treatment period. Patients are classified as a treatment failure if they meet one of the following criteria:

i. A 50% increase in the frequency of paroxysms compared to the final 7 days of the open-label period to more than three paroxysms within a 7-day period

ii. When more than three paroxysms are reported in a 7-day period, a 50% increase in the severity of pain experienced in the paroxysms compared to the final 7 days of the open-label period

iii. A Patient Global Improvement of Change rating of much worse/very much worse

iv. The patient discontinues the study because of ‘lack of efficacy.’

v. The patient discontinues because of an adverse reaction or poor tolerability considered to be related to the study medication

**Figure 2:**
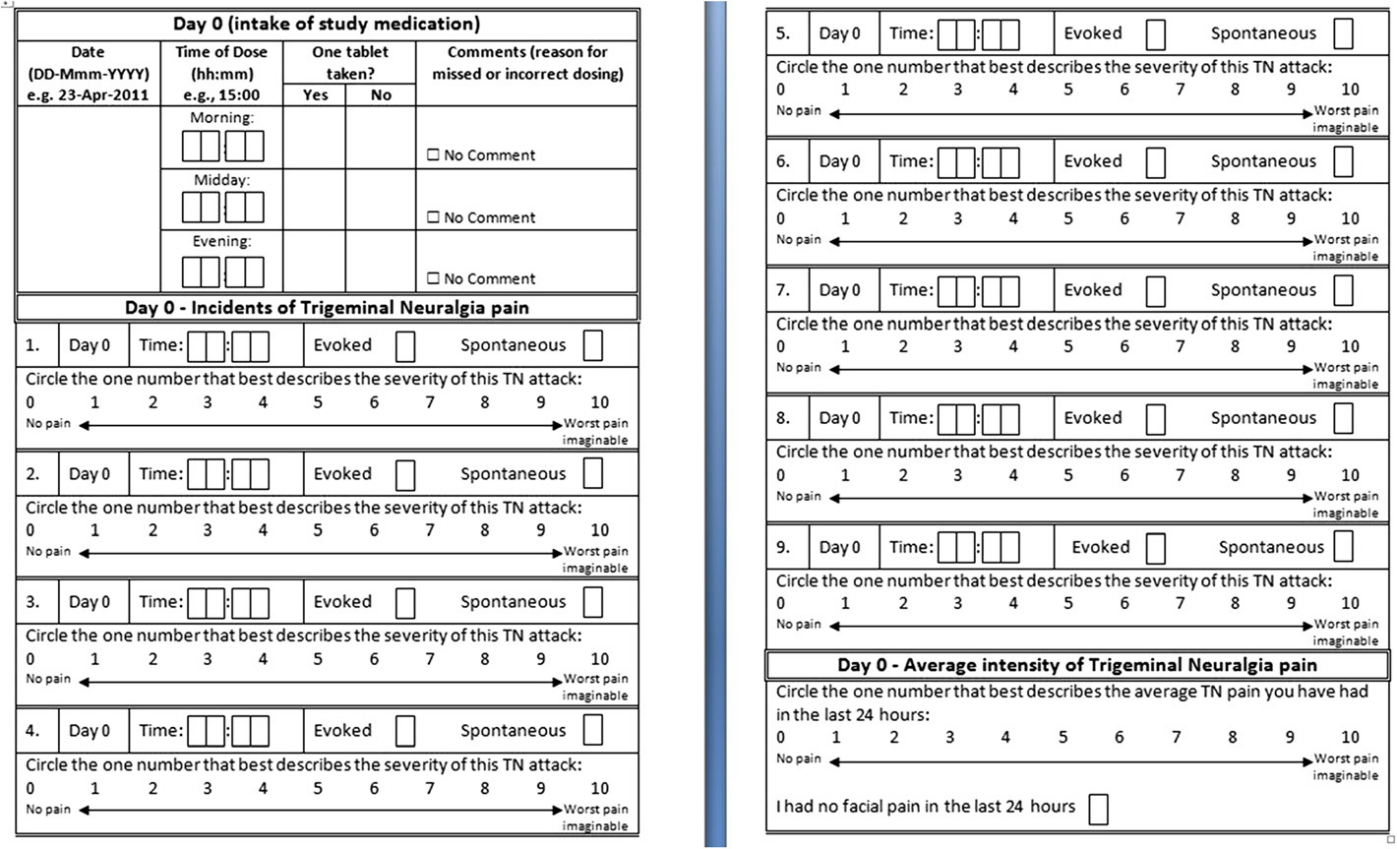
Pain diary completed by the patient on a daily basis.

During the double-blind randomized phase, patients will be evaluated to determine whether they meet the failure criteria at each clinic visit, which will occur every 7 days in the double-blind treatment period. The data for the frequency and severity of the pain will be taken from the daily diaries completed by the patients. Figure 2 is an example of such a diary.

Secondary outcome measures of efficacy are:

• Proportion of failures by week

• Kaplan-Meier analysis of time to failure

• Average change in the pain intensity numerical rating scale PI-NRS by week

• Average change in PI-NRS (best response)

• Median change in the number of paroxysms by week

• Median change in the number of paroxysms (best response)

• Patient and physician global impression of change

• Average change in the Brief Pain Inventory BPI-Facial by week [35]

• Average change in the BPI-Facial (best response)

• Number and severity of paroxysms of pain in the 21-day open-label period, both evoked and spontaneous

• Average 24-h pain intensity numerical rating scale (PI-NRS)

• Patient and Physician Clinical Global Impression of Change

Other measures will include:

• Adverse events, vital signs, ECG and safety laboratory samples will be collected throughout the study

• Adverse event profile: this questionnaire was developed for epilepsy to understand the common AEs associated with epilepsy [36,40], collecting it at screening, day 21 and the last day in the study to try and compare tolerability on CNV1014802 compared to other anti-epileptics they may be taking at screening.

• Medical Outcomes Study-Cognitive Scale (MOS-Cog) will be completed at screening and the end of the study [38,39]

• Assessment of blindedness of study medication: Both the patient and clinician will provide a guess as to which medication (CNV1014802 or placebo) was administered during the double-blind treatment period

• Pre- and post-dose plasma concentrations of CNV1014802

• Presence of genetic mutations in sodium channel (Nav1.1, Nav1.2, Nav1.3, Nav1.6 and Nav1.7) or calcium channel (Cav2.2 and Cav2.1) genes

If the patient requires a drug treatment for their TN pain other than paracetamol, they will be considered a treatment failure, also if they develop any of the following: seizure or suspected seizure, elevated hepatic transaminases, QT prolongation, drug-related rash, and moderate to severe CNS adverse events.

### Inclusion and exclusion criteria

All patients are aged 18 to 80 years of any gender. The main inclusion and exclusion criteria are shown in Table 1; the others are listed on the website http://www.clintrials.gov, trial no. NCT01540630.

**Table 1:** CNV1014802 in patients with trigeminal neuralgia inclusion and exclusion criteria

***Major inclusion criteria***	***Major exclusion criteria***
A patient will be eligible for inclusion in this study only if all of the following criteria apply:	A patient will not be eligible for inclusion in this study if any of the following criteria apply:
1. The following diagnostic criteria for trigeminal neuralgia must be met:	1. Patients who are known non-responders to sodium channel blockers at therapeutic doses. If patients have previously been unable to tolerate sodium channel blockers and therefore has not been able to take doses within the therapeutic dose range, they may still be included.
Paroxysmal attacks of pain lasting from a fraction of a second to 2 min affecting one or more divisions of the trigeminal nerve	
Pain has at least one of the following characteristics:	
i. Intense, sharp, superficial or stabbing	
ii. Precipitated from trigger areas or by trigger factors	
iii. Attacks are stereotyped in the individual patient	
There is no clinically evident neurological deficit	
Not attributed to another disorder	
2. Frequency criteria for numbers of paroxysms:	2. A positive history of HIV.
Patients must have suffered a minimum of 3 or more paroxysms of pain per day, rated at an intensity of 4 or more on the pain NRS, on at least 4 days during the last 7 days prior to entry into the open-label treatment period.	
3. Male or female between 18 and 80 years of age inclusive at the time of signing the informed consent.	3. A positive pre-study hepatitis B surface antigen or positive hepatitis C antibody result within 3 months of screening.
4. A female patient is eligible to participate if she is of non-childbearing or child-bearing potential and agrees to use one of the contraception methods listed.	4. History of any liver disease within the last 6 months, with the exception of known Gilbert’s disease.
5. Male patients must agree to use one of the contraception methods.	5. History of excessive regular alcohol consumption within 6 months of the study defined as: an average weekly intake of >28 units or average daily intake >4 units for males; an average weekly intake >21 units or average daily intake >3 units for females. One unit is equivalent to 8 g of alcohol: a half-pint (~240 ml) of beer, 1 glass (125 ml) of wine or 1 (25 ml) measure of spirits.
6. Body weight ≥ 50 kg for men and ≥ 45 kg for women.	6. Patients with a history or risk of seizures or a history of epilepsy, head injury or related neurological disorders.
7. BMI ≤34.9.	7. Patients with a history of uncontrolled or poorly controlled hypertension.
8. Capable of giving written informed consent, which includes compliance with the requirements and restrictions listed in the consent form. Informed consent must be obtained prior to the commencement of any study-related procedures.	8. History or presence of significant cardiovascular, gastrointestinal, or renal disease or other condition known to interfere with the absorption, distribution, metabolism or excretion of drugs, which, in the opinion of the investigator, may interfere with the study procedures or compromise patient safety.
	9. Patients with conditions known to affect cardiac conduction or a personal or familial history of Brugada syndrome.
	10. Females of child-bearing potential only: pregnant females as determined by positive urine or serum hCG test at screening or prior to dosing.
	11. Lactating females.
	12. History or presence of any clinically significant abnormality in vital signs/ECG/laboratory tests, or any medical or psychiatric condition, which, in the opinion of the investigator, may interfere with the study procedures or compromise patient safety.
	13. The patient has a history of suicidal ideation and/or suicide attempts.
	14. The patient has clinical evidence of recent major depression (by patient’s medical history).

Patients must have classical trigeminal neuralgia fulfilling all the criteria of the IHS [13]. They must have suffered a minimum of three or more paroxysms of pain per day, rated at an intensity of 4 or more on the pain NRS, on at least 4 days during the last 7 days prior to entry into the open-label treatment period. The following patient categories may be considered for the study: suboptimal responders or intolerant to sodium channel blockers, responders to sodium channel blockers who are willing to be washed out from any sodium channel blockers prior to the run-in period, suboptimal responders to pregabalin or gabapentin, recurrence of trigeminal neuralgia following a remission including (but not limited to) patients who have previously had a positive response to surgery, treatment naïve and on waiting list for surgery.

It is considered useful for all patients to have had some form of imaging done to exclude symptomatic TN and also to have had a dental examination to exclude any potential dental causes. Patients with significant autonomic symptoms or other neuropathic pain are to be excluded.

In view of the fact that CNV1014802 is extensively metabolized, and that the drug interaction potential in humans has not yet been fully evaluated, a variety of drug types have to be stopped prior to study commencement, and approved concomitant medications must have been stable for at least 3 weeks prior to day 0. This includes but is not limited to sodium channel blockers or drugs that adversely interact with a monoamine oxidase-B inhibitor: MAOIs, antidepressants, opioids and sympathomimetic agents. Other than gabapentin and pregablin, no other antiepileptic drug (AED) is allowed.

Among the exclusion criteria are patients who are known non-responders to sodium channel blockers at therapeutic doses and those with a history of uncontrolled or poorly controlled hypertension. Patients with a history or presence of significant cardiovascular, gastrointestinal or renal disease or other condition known to interfere with the absorption, distribution, metabolism or excretion of drugs are also excluded.

The rescue medication is paracetamol only to a maximum dose of 4 g/day.

As the trial is intended to be carried out in multiple countries, it is crucial to ensure that only classical TN patients are recruited and not those whose diagnosis is equivocal and who had tried a large number of drugs with poor response. The data-monitoring committee (DMC) is made up of three clinical investigators with experience in diagnosing patients with TN (JZ, DE, MO), the medical director and study manager from Convergence and from the CRO medical monitor, statistician and project leader. Their remit as well as verifying the diagnosis is to evaluate ongoing efficacy and safety data from the open-label period and to recommend: the dose of CNV1014802 to be used following review of the first ten evaluable subjects completing the open-label period. All patients considered for screening are required to complete a two-page form on diagnostic criteria to be considered by the DMC and approved by at least two of the investigators. The DMC also receives weekly reports on the progress of the trial and numbers required from each center and their progress through the study. It can also terminate the study depending on the efficacy and tolerability of the drug.

All the data from the trial are collected on electronic clinical record forms and anonymized prior to submission, and each site is visited by the project lead and local lead from the CRO. All investigators must adhere to Good Clinical Practice (GCP) and have undergone the appropriate training. All the investigational site staff took part in the protocol training program, either face to face or web based.

The study is double-blind and there are strict restrictions on unblinding. The investigator or treating physician may unblind a patient’s treatment assignment only in the case of an emergency, when knowledge of the study treatment is essential for the appropriate clinical management or welfare of the patient.

Pharmacovigilance staff acting on behalf of the sponsor may unblind the treatment assignment for any patient with a serious adverse event (SAE). If the SAE requires that an expedited regulatory report be sent to one or more regulatory agencies, a copy of the report, identifying the patient’s treatment assignment, may be sent to clinical investigators in accordance with local regulations.

### Power calculation

The aim is to enroll sufficient patients to randomize 30 responders into the double-blind randomized phase of the study. If evaluable data are available from 22 patients entering the randomized phase, assuming a 20% failure rate for CNV1014802 and a 67% failure rate for placebo, 11 evaluable patients per treatment arm (22 in total) would provide 80% power to detect a difference of 47% between the proportion of failures on CNV1014802 and placebo, assuming a one-sided test with a type I error rate of 5%.

## Discussion

This is the first time that such a design has been used to evaluate a drug for TN. It is also the first time that a drug is being used that has not first been evaluated in epilepsy. Its design conforms with the CONSORT guidelines, which have been shown to improve the quality of trial reporting [41], and although conceived before the statement on defining the standard protocol for clinical trials was published, it does conform to these [26]. Drug trials in TN are rare, heterogeneous and often contain serious methodological issues. Only 11 randomized placebo controlled trials on classical TN have been conducted since 1967. These trials recruited very different patient numbers (ranging from 3 to 77 participants), often embedding TN patients into a broader facial pain patient spectrum study to gain more statistical power. Formal power calculations were generally not provided in most previous TN trials. The study by Gilron et al. [42] is an extreme example of recruitment difficulties that all TN trials face. This placebo-controlled, multiple cross-over pilot study included only three patients. The present study is also small and needs to be regarded as a pilot, which will enable more accurate power calculations to be performed in the future. The primary outcome measure chosen is pain relief as is the norm for pain trials, yet it may be that other outcomes such as improved quality of life due to reduced side effects may be of greater importance. Many patients report that the “odd twinge” does not significantly impact on their lives, but tiredness, impaired cognitive function and ataxia from the high-dose medication has a significant impact on activities of daily living.

Most centers specialized in the treatment of patients with TN do not have a large enough patient base of the right type of patients including those willing to have their medication regimens changed.

A variety of strategies has been suggested to increase recruitment to trials of both participants and clinicians, and a recent systematic review suggests increased education of clinicians about the benefits of RCTs is needed [43]. Thus, the sites chosen have a special interest in TN with experienced clinicians, and it is hoped they will be able to recruit the appropriate patients. It takes time to recruit patients to clinical trials, and there are few data available on this topic. A recent study of an orthodontics multicenter RCT showed that it took on average 19 min to recruit a patient to the study and then a further 110 min per patient to fully recruit to the study and ensure all administrative data were available [44]. The variations between the centers were large in respect to the administrative data time as it often depended on the research support staff that was available. There was also considerable time involved in meetings of the principal investigators, and the authors suggest that these timings are potentially much higher in more complex studies.

To improve recruitment to this trial ethics approved data for patients were prepared and distributed to clinicians attending relevant conferences and for placement in a Trigeminal Neuralgia Support group newsletter in the UK. Recruitment of patients could be problematical for other reasons that are specific to this condition. TN causes very severe pain, and once stabilized on medications patients are often reluctant to change for fear their pain will return or become unmanageable. They may have used a range of other AEDs including the two permitted ones in the past and so be reluctant to go back to them. Currently the most effective AEDs cannot be used. Patients with classical TN who have neurovascular compression of the nerve (seen on MRI) may opt to have an operation rather than try further medications as the outcomes from surgery can be better [15]. The population most at risk is the elderly, and they are likely to have medical conditions that preclude their inclusion in the trial.

Different disease definitions as well as imprecise allocation of non-standardized diagnostic criteria make comparison of the different patient populations virtually impossible. Many trials do not refer to any particular diagnostic criteria at all but are content to describe their patient as having classical TN without further clarification or definition of that term. This study uses the international classification of headache disorders (ICHD-3beta) and an experienced data management committee to ensure a standardized patient selection and prevent non-classical TN patients entering the trial.

Incomplete or insufficiently defined outcome parameters, which were not clearly stated in many of the RCTs conducted until now, is another serious problem. Many trials failed to report dropouts or withdrawals, while those that did report these measures did not use intention-to-treat analysis to analyze their study results. This is particularly problematic since the dropout and withdrawal rates in most TN trials are very high. Information on demographics and clinical characteristics of the different treatment groups is often incomplete and lacks a clear comparison of baseline data with regard to gender, age, duration of disease and pain scores.

Allocation concealment and blinding are often problematic in randomized controlled trials on TN. Often blinding methods are not reported in detail, so that it remains unknown who was blinded to what. All of these trials stated that they were randomized, but most did not describe the utilized method of randomization or whether the randomization method used was effective in regard to homogenizing the groups that were to be compared in the final analysis.

Selective reporting is generally considered a minor problem in these small and often confined studies. Some studies however did not clearly state what assessment tools they used for their analysis, while others did not report the adverse events or side effects of the study drug consistently. The possibility of patients going into spontaneous remission with treatment or without was not raised by most authors when interpreting their data.

Other potential sources of bias were often underreported. Many studies use the investigated drug as add-on to carbamazepine or other established treatment regimens [34,43,45-49]. Considering the pharmacologic profile of carbamazepine and other frequently used antiepileptic drugs with many drug interactions and hepatic metabolism, this may also pose serious consequences to the trial outcome and data interpretation of these trials that we are only starting to marginally comprehend. One of the largest RCTs comparing carbamazepine and oxcarbazepine in 46 patients has only been reported as a conference abstract, and the other study, possibly the same one in a German journal, included 48 patients [50]. The EERW design is new, and there will be few trials against which to compare the results, but the outcome measures being used are from the range of those suggested by Dworkin et al. [33]. The recently extended Brief Pain Inventory [35] has been psychometrically tested but has not yet been tested in terms of sensitivity to change; however, the first section has been used in extensive analgesic trials and so outcomes can be compared.

This protocol is being published prior to the study being completed in accordance with the recent literature [51] so that it will be possible to compare what was intended and what was actually done, and it has been registered on a trials register since February 2012, which may help to recruit patients. A comparison of registered and published primary outcome measures in RCTs shows selective outcome reporting is still prevalent [52], but this may also be due to an incorrect choice of outcome measures. In this study it is difficult to determine how large a change can be expected as there are currently so few data in the RCTs on TN, and unlike other pain trials, 100% pain relief can be achieved as opposed to the usual 50%.

The results of the trial will be published and presented at conferences including those for patients.

## Trial status

Ongoing

## Abbreviations

AED: Antiepileptic drugs; AE: Adverse event; CBZ: Carbamazepine; Bid: Twice daily; CRO: Contract research organization; EERW: Enhancement enriched randomized withdrawal designs; ICHD: International classification of headache disorders; DB: Double blind; DMC: Data monitoring committee; MHRA: Medicines and healthcare products regulatory agency UK; RCT: Randomized controlled trial; Tid: Three times daily; TN: Trigeminal neuralgia; SAE: Serious adverse event.

## Competing interests

JZ, DE and MO received payments for consultancy work on the data monitoring committee. KG, JP, GG, VM and ST are full-time employees of Convergence Pharmaceuticals, Ltd. The authors declare that they have no competing interests.

## Authors’ contributions

JZ participated in the design and coordination, was head of the data monitoring group and drafted the paper. DE and MO participated in the design of the data monitoring group, monitored the whole trial from the clinical standpoint and contributed to drafting the paper. KG and JP conceived the study, participated in the design and coordination, and drafting of the paper. GG, VM and ST were responsible for the chemistry and pharmaceutical development, preclinical studies and electrophysiological characterization of CNV1014802 and the drafting of the paper. All authors read and approved the final manuscript.

## Authors’ informations

JZ is the current head of the largest UK multidisciplinary facial pain unit and a pain medicine specialist with a particular interest in trigeminal neuralgia, conducted both medical and surgical trials and Cochrane author of systematic reviews on trigeminal neuralgia. JP is Head of Clinical Operations at Convergence Phamaceuticals, Ltd. DE is the Head of the Interdisciplinary Orofacial Pain Unit of the University of Zurich, Switzerland. MO is the scientific head of the West-German Headache Center and consultant neurologist at the Department of Neurology at the University Hospital Essen, University of Duisburg-Essen. GG is Head of Chemistry and Preclinical Development at Convergence Pharmaceuticals, Ltd. VM is Head of Electrophysiology at Convergence Pharmaceuticals, Ltd. ST is Chief Scientific Officer at Convergence Pharmaceuticals, Ltd. KG is Medical Director at Convergence Pharmaceuticals, Ltd.

## Supplementary Material

Additional file 1 TGN expert summary Dec 2010. Click here for file

Additional file 2 Ethics committees. Click here for file
