# Supplementary material for: Novel design for a phase IIa placebo-controlled, double-blind randomized withdrawal study to evaluate the safety and efficacy of CNV1014802 in patients with trigeminal neuralgia
Source: Trials. 2013 Nov 23;14:402. doi: 10.1186/1745-6215-14-402 (PMC4222641; doi:10.1186/1745-6215-14-402)
Supplement: Additional file 1 — TGN expert summary Dec 2010. [file 1745-6215-14-402-S1.doc]

**Additional file 1**

**Trigeminal Neuralgia; Summary of Discussions with Experts**

A series of TC’s were held with Joanna Zakrzewska (JZ; London), Jeffrey Cohen (JC; New York) and Zaza Katsarava (ZK; Essen) during December 2010. A proposal for a randomised withdrawal TGN PoC trial was pre-circulated to the experts, following initial discussions with JZ. A pdf copy is attached.

The clear consensus was that the study as proposed was not viable, without some changes. The key issues that needed to be addressed are summarised below:

**Washout Phase**

Patients with TGN experience severe paroxysms of pain and are not willing to be washed out from medication, which is most likely to be sodium channel blockers (carbamazepine or oxcarbazepine). Current recommendations with CNV1014802 are that all sodium channel blockers must be washed-out prior to the start of 802, because of the risk of adverse pharmacodynamic interaction. Adverse PD interactions (mainly CNS AE’s) have been observed in the absence of PK interaction following the co-administration of lamotrigine with carbamazepine (Besag 1998).

All experts recommended that either co-administration of sodium channel blockers with 802, or prefereably a rapid down-titration of existing medication with initiation of 802 (either at full dose or by titration) would be required to make a study viable. However, such an approach would increase the possibility for CNS AE’s to be experienced and attributed to 802. This could potentially influence the benefit risk profile of the compound.

Other possibilities discussed included moving patients from their sodium channel blockers onto a gabapentinoid (gabapentin or pregabalin), and then allowing introduction of 802. This again has potential issues, including potential PD interaction (primarily CNS AE’s which are common with the gabapentinoids), and the patient acceptability of altering medications with risk of exacerbation of pain.

The use of treatment-naive patients was briefly discussed, although due to the scarity of such patients it was dismissed as non-viable.

The experts concurred that a washout period in TGN is not feasible, and any protocol would need to take into account the marked reluctance of patients to change medication regimes for short-term benefit in the proposed study.

**Inclusion Criteria**

There was a general consensus to use the IHS criteria for TGN. It was recommended to have a small committee to review individual patients diagnosis before inclusion into the trial, to ensure diagnostic homogeneity. It was agreed surgery failures could be included. Patients with continuous facial pain (but not those with post-paroxysmal pain) should be excluded. ZK suggested stratifying patients into those with and without concomitant facial pain.

There was some uncertainty over the number of paroxysms of pain per day as an entry criterion. ZK felt 4/day could be too high.

Age restrictions were of clear concern. An upper age limit of 65 years is likely to make the study non-viable (ZK); an upper limit of 70 was seen as the minimum necessary, due to the age of the presenting patients. This is a clear risk for 802 as in the absence of elderly PK data, regulatory agencies may put an upper age limit of 65 years on clinical trials.

**Study Endpoints**

The experts were generally supportive of the proposed endpoints. However, ZK ‘s opinion was that a 50% response rate in number of attacks was too ambitious in the open period; his recommendation was for a 30% response. JZ and JC suggested that spontaneous attacks were more likely to reduce than evoked attacks. In the DB period, JZ recommended that safety dropouts should also be termed treatment failures.

Other suggestions for endpoints included some assessment of cognitive function (JZ).

**Recruitment and Operational**

All experts re-iterated the scarcity of patients, and the fact that patients are reluctant to alter their medication regimens. Both JZ and ZK have relatively large cohorts of patients. Although realistic recruitment rates are hard to define in the proposed trial, an estimate of 3-4 patients per site per year was regarded as likely (subject to a study design based around the current proposal).

It was suggested that a major impediment to recruitment is the non-availability of compassionate 802 for treatment responders. JZ mentioned that ethics committees might ask for this.

The DDI potential for 802 was another likely hindrance to recruitment, especially if statins were to be excluded.

**Conclusion**

A randomised withdrawal study represents the most viable design for PoC in TGN, but there are considerable risks in running such a study. A workable study with 802 would require some degree of initial concomitant administration with other sodium channel blockers, with consequent risk of adverse PD interactions. The study would require a relatively large number of TGN patients (we have assumed minimum of 30 randomised), and such a study has never been previously performed in this indication. Most recent TGN trials have had major issues with recruitment. The potential age restriction with 802 of 65 years represents another substantial risk.

It is likely that the proposed study would require some 10-12 centres in Western Europe/North America to recruit over a sustained period. Even then, risk of sub-optimal recruitment cannot be excluded. There are also cost and resource issues with this approach. A potential solutions to could include running the study in countries with poorly treated TGN patients (such as South America or SE Asia), but this carries a high level of risk, and potential expense.

In summary, whilst there is a good potential up-side in running a successful PoC study in TGN, recent consultations have highlighted some substantial risks for 802 and for Convergence.

**Study Proposal PDF**
