# Supplementary material for: Novel design for a phase IIa placebo-controlled, double-blind randomized withdrawal study to evaluate the safety and efficacy of CNV1014802 in patients with trigeminal neuralgia
Source: Trials. 2013 Nov 23;14:402. doi: 10.1186/1745-6215-14-402 (PMC4222641; doi:10.1186/1745-6215-14-402)
Supplement: Additional file 2 — Ethics committees. [file 1745-6215-14-402-S2.doc]

Additional file 2

Ethics Committee names which approved study Phase IIa Placebo-Controlled, Double-Blind Randomized Withdrawal Study to Evaluate the Safety and Efficacy of CNV1014802 in Patients with Trigeminal Neuralgia

1.NRES Committee East of England, UK - for three centres

2. Ethik-Kommission Medizinische Fakultät der Universität Duisburg-Essen, Germany

3. De Videnskabsetiske Komiteer for Region Hovedstaden, Denmark

4. IRCCS Az. Osp. Un. San Martino -IST Ist. Naz. Per la Ricerca Sul Cancro, Italy

5. Azienda Policlinico Umberto I, Italy

6. Kantonale Ethikkommission Zürich (KEK), Switzerland

7. Ethics Committee of the University of the Free State, South Africa
8. Comité de Protection des Personnes Sud-Méditerranée V, France

9.Comité Ético de Investigación Clínica - Área 2, Spain

10.Research Ethics Committee of the University of Tartu, Estonia

11.Clinical Research Ethics Committee at Pauls Stradins, Latvia

12.Lithuanian Bioethics Committee, Lithuania

13. National Ethics Committee for Clinical Study in Medicine, Romania
